# Transcranial Photobiomodulation for Spasticity in Pediatric Cerebral Palsy: A Scoping Review of Neurodevelopmental Considerations, Treatment Protocols, Functional Outcomes, and Methodological Gaps

**DOI:** 10.3390/brainsci16030272

**Published:** 2026-02-28

**Authors:** Amalio Jiménez, Frederick R. Carrick, Monèm Jemni

**Affiliations:** 1 The Carrick Institute, Cape Canaveral, FL 32920, USA; hfcpr97@hotmail.com (A.J.); drfrcarrick@post.harvard.edu (F.R.C.); 2 Centre for Mental Health Research in Association, The University of Cambridge, Cambridge CB2 1TN, UK; 3 College of Medicine, University of Central Florida, Orlando, FL 32827, USA; 4 Burnett School of Biomedical Science, University of Central Florida, Orlando, FL 32827, USA; 5 Department of Health Professions Education, MGH Institute for Health Professions, Boston, MA 02129, USA; 6 Faculty of Physical Education, Ningbo University, Ningbo 315000, China

**Keywords:** photobiomodulation, low-level laser therapy, cerebral palsy, spasticity, pediatric, scoping review, transcranial, neuromodulation

## Abstract

**Highlights:**

**What are the main findings?**

- The current evidence base for transcranial photobiomodulation (tPBM) in pediatric cerebral palsy spasticity is critically small (only five studies), methodologically weak, and characterized by profound heterogeneity and inconsistent dosimetry reporting.
- Despite reported reductions in spasticity, the absence of sham-controlled, blinded trials and pediatric-specific safety protocols prevents any attribution of these effects to tPBM rather than placebo or other factors.

**What are the implications of the main findings?**

- tPBM must be considered an unproven experimental intervention and should not be used clinically for pediatric CP spasticity outside of rigorously controlled research trials.
- Future research must prioritize foundational, sham-controlled, dose-ranging trials based on pediatric-specific computational head models and standardized safety and dosimetry reporting.

**Abstract:**

*Background:* Spasticity, affecting over 80% of children with cerebral palsy (CP), is a major source of disability. Transcranial photobiomodulation (tPBM) is a promising non-invasive neuromodulatory intervention, but evidence for its use in pediatric CP spasticity is fragmented and limited. Objective: This scoping review aimed to systematically map the literature to characterize neurodevelopmental considerations, treatment protocols, functional outcomes, and methodological gaps related to tPBM for spasticity in pediatric CP. *Methods:* The review followed the PRISMA extension for Scoping Reviews (PRISMA-ScR). A systematic search was performed across eight databases from January 2000 to September 2025. The PCC framework guided inclusion of studies involving children/adolescents (0–18 years) with CP and spasticity, investigating PBM with a primary focus on transcranial application. *Results:* From 345 records, only five primary studies (total *n* = 45 children) met the inclusion criteria. The evidence base is severely limited and heterogeneous, with only two of the five studies applying tPBM. A profound lack of protocol standardization was identified, with frequent reports that are internally inconsistent or contain physically implausible dosimetry reporting. Methodological quality was pre-preliminary, with no sham-controlled or adequately blinded trials. Although all studies reported reductions in spasticity and/or motor improvements, the high risk of bias prevents attribution of the effects to tPBM. *Conclusions:* The evidence supporting tPBM for spasticity in pediatric CP is in a pre-preliminary stage, defined by a critically small number of studies, a pervasive lack of methodological rigor, and an absence of pediatric-specific safety protocols. The field requires an immediate pivot to foundational, sham-controlled, double-blind trials with standardized dosimetry before tPBM can be considered anything other than an unproven experimental intervention.

## 1. Introduction

Cerebral palsy (CP) is the most common cause of childhood motor disability, arising from non-progressive disturbances in the developing brain [1]. Spasticity, classically defined as a velocity-dependent increase in tonic stretch reflexes, is a common and impactful impairment affecting over 80% of children with CP [2]. Contemporary understanding, however, frames motor dysfunction in CP within the broader, multidimensional concept of “muscle overactivity,” which includes spasticity, co-contraction, dystonia, and secondary soft tissue changes like contractures, coupled with weakness and loss of selective motor control [2,3]. This review uses “spasticity” as the primary search and outcome term due to its historical use in the literature, but it is acknowledged that the reported outcomes may reflect changes in this broader spectrum of neural and peripheral contributors to hypertonia. Standard treatments, including physical therapy, oral antispasmodics, botulinum toxin injections, and neurosurgery, are variably effective but can be invasive, have systemic side effects, or provide only transient benefits [4,5]. This context motivates investigating safe, non-invasive, biomodulatory adjuncts like transcranial photobiomodulation (tPBM). Unlike invasive or pharmacologic interventions that often target symptoms locally, tPBM is a non-invasive neuromodulatory approach with the potential to address underlying neuropathophysiology, such as mitochondrial dysfunction and neuroinflammation [6,7]. Its potential for a systemic, non-pharmacological effect and a favorable safety profile provide a compelling rationale for its investigation, even amidst a currently weak evidence base [8,9].

Transcranial photobiomodulation involves applying low-power, high-fluency red or near-infrared light (600–1100 nm) to the scalp to modulate neurobiological functions without thermal damage. The primary molecular chromophore is cytochrome c oxidase (CCO), a key enzyme in the mitochondrial electron transport chain [10,11]. Photon absorption dissociates inhibitory nitric oxide (NO) from CCO, leading to increased electron transport, oxygen consumption, and ATP production [11]. Secondary mechanisms include the transient release of reactive oxygen species (ROS) at sub-toxic levels and cyclic AMP (cAMP), which activate transcription factors such as NF-κB and CREB [6,7]. This signaling cascade upregulates genes related to cell survival, antioxidant defenses, anti-inflammatory cytokines, and neurotrophic factors (e.g., brain-derived neurotrophic factor, BDNF). The net effects are enhanced mitochondrial function, reduced neuroinflammation, increased neuroprotection, and promotion of synaptic plasticity [6,7,12]. This mechanistic plausibility has spurred interest in tPBM as a novel neuromodulatory approach for conditions like CP [13].

The field of tPBM for pediatric CP spasticity is characterized by extreme heterogeneity in treatment protocols, small and uncontrolled study designs, and a fragmented evidence base. A scoping review is the most appropriate methodology to systematically map this emerging and complex literature [14]. It allows for the characterization of the range of existing interventions, the identification of key concepts and outcomes, and the clarification of critical methodological gaps, without assessing the risk of bias or efficacy in a manner more typical of a systematic review. This approach is essential to provide a foundational map to guide future, more standardized research.

**Aim and Objectives**
**Aim:** To systematically map and critically appraise the existing evidence on transcranial photobiomodulation for the management of spasticity in children and adolescents with cerebral palsy.
**Objectives:**
  1. To identify and characterize the tPBM treatment protocols used, including parameters, application sites, and dosimetry.
  2. To summarize the reported effects on spasticity and functional outcomes.
  3. To map the methodological quality of the evidence base and identify critical gaps, particularly regarding neurodevelopmental considerations and safety reporting.
  4. To provide recommendations for future research based on the synthesized gaps.

### Molecular Mechanisms of Photobiomodulation: A Foundation for Neuromodulation

The biological rationale for tPBM is grounded in its well-characterized molecular and cellular effects. The primary chromophore is cytochrome c oxidase (CCO), the terminal enzyme in the mitochondrial electron transport chain. Absorption of red/NIR light photons by CCO leads to the photo-dissociation of inhibitory nitric oxide (NO), enhancing electron transport, oxygen consumption, and ATP synthesis 10, 11. This boost in cellular energy metabolism is considered the foundational photobiomodulation event [15,16].

This primary mitochondrial response triggers a cascade of secondary signaling pathways. A transient, sub-toxic increase in reactive oxygen species (ROS) and the release of cyclic AMP (cAMP) act as signaling molecules that activate key transcription factors, including NF-κB and CREB 6, 7. The subsequent upregulation of gene expression drives the synthesis of:

Neurotrophic factors (e.g., brain-derived neurotrophic factor, BDNF), which are crucial for neuronal survival, axonal growth, and synaptic plasticity [17,18].

Anti-inflammatory cytokines (e.g., IL-10) and anti-apoptotic proteins, reducing chronic neuroinflammation [19,20].

Endogenous antioxidant enzymes (e.g., superoxide dismutase), restoring redox balance [21,22].

At a system level, these interconnected effects, enhanced mitochondrial bioenergetics, attenuated neuroinflammation, and promoted plasticity, provide a compelling mechanistic framework for tPBM investigation in CP. Theoretically, by improving metabolic function in cortical and spinal motor circuits, reducing glial-mediated inflammation, and facilitating experience-dependent synaptic reorganization, tPBM could address several core pathophysiological components of CP-related motor dysfunction.

## 2. Methods

This scoping review was conducted in accordance with the Preferred Reporting Items for Systematic Reviews and Meta-Analyses extension for Scoping Reviews (PRISMA-ScR) [14,23]. A pre-defined protocol outlined the objectives, search strategy, and data analysis plan.

### 2.1. Eligibility Criteria (PCC)

- **Population:** Children and adolescents (age 0–18 years) with any type of cerebral palsy where spasticity was a reported characteristic.
- **Concept:** Interventional strategies utilizing photobiomodulation (PBM), with a primary analytical focus on transcranial PBM (tPBM). Peripheral PBM applications were included solely for contextual benchmarking.
- **Context:** All clinical and research settings. All original study designs (e.g., randomized controlled trials, case series, and experimental studies) were eligible.
- **Exclusion criteria:** Reviews, commentaries, editorials, animal studies, and studies not published in English. Non-English studies were excluded for pragmatic reasons, a decision supported by evidence that this does not typically alter systematic review conclusions [24].

### 2.2. Information Sources and Search Strategy

A systematic search was performed across eight electronic databases: PubMed, Cochrane CENTRAL, Scopus, Web of Science, Embase, SciELO, Dialnet, and Google Scholar. The search strategy combined Medical Subject Headings (MeSHs) and keywords related to: (1) photobiomodulation (e.g., “transcranial photobiomodulation” and “low level light therapy”), (2) pediatric cerebral palsy, and (3) spasticity or motor outcomes (e.g., “spasticity” and “Modified Ashworth”). The search covered studies published from 1 January 2000 to 30 September 2025. This timeframe captures the modern conceptual and technological era of PBM research relevant to neuromodulation.

### 2.3. Selection of Sources of Evidence

**Figure 1:**
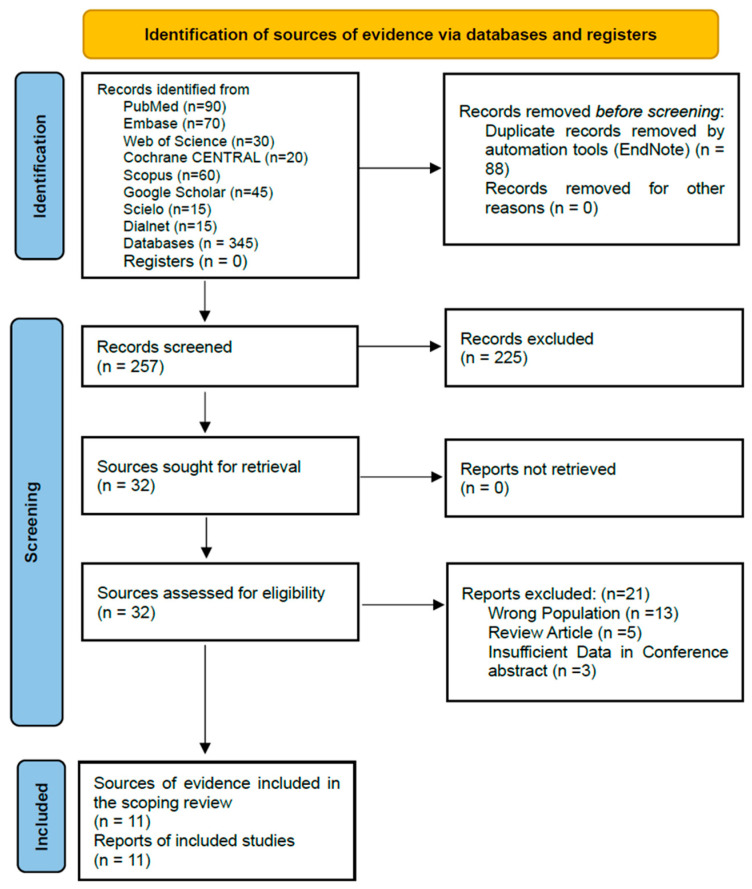
PRISMA-ScR flow diagram of the study selection process.

The initial search yielded 345 records. After duplicate removal, 257 unique records were screened by a single reviewer based on titles and abstracts. To enhance rigor, a one-week interval was observed between screening phases, and all exclusion reasons were documented. Subsequently, 29 full-text reports were assessed for eligibility, resulting in five studies included in the final synthesis. The study selection process is detailed in the PRISMA flow diagram (Figure 1).

### 2.4. Data Extraction (Charting)

A standardized data extraction form was developed. Key variables included: study and participant characteristics, PBM intervention parameters (wavelength, power, energy density, site, and sessions), outcome measures (e.g., Modified Ashworth Scale and Gross Motor Function Measure), key findings, and adverse events. The data extraction template is provided in the Supplementary Materials.

### 2.5. Data Analysis and Synthesis

The analysis involved a descriptive numerical summary of the study characteristics and a narrative synthesis focused on mapping the intervention parameters, outcomes, and methodological features. Data was tabulated to illustrate patterns and heterogeneity.

### 2.6. Critical Appraisal and Ethical Considerations

Consistent with scoping review methodology, a formal risk-of-bias assessment was not conducted. Instead, the review explicitly maps methodological features, reporting quality, and the internal consistency of dosimetry. As a review of the published literature, formal ethical approval was not required.

## 3. Results

### 3.1. Study Selection

The PRISMA flow diagram (Figure 1) outlines the selection process. From 345 initial records, 88 duplicates were removed. Screening of 257 titles and abstracts excluded 228 records. Full-text assessment of 29 articles led to the exclusion of 24, resulting in five primary studies included in the final synthesis (Figure 1. PRISMA flowchart 2020 diagram).

### 3.2. Characteristics of Included Studies

The characteristics of the five included studies are summarized in Table 1. The evidence base is critically small (total *n* = 45 children from clinical studies) and heterogeneous. Notably, only two of the five studies applied PBM transcranially [25,26], with the majority utilizing peripheral application [27,28,29]. Study designs were predominantly uncontrolled observational or pilot studies, with frequent omissions of basic demographic data like age and sex. The temporal distribution of these studies (2016 onward) underscores the nascent state of this specific clinical research area.

**Figure 2:**
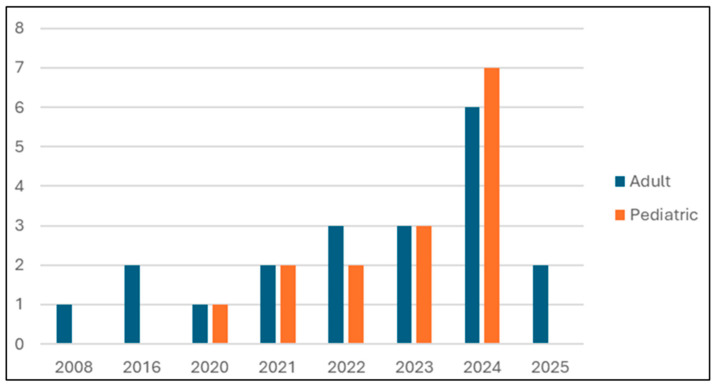
Temporal distribution of included studies from 2008 to 2025. Note: Compares pediatric population-focused research (red) with adult/general mechanisms/reviews (blue). A marked increase in pediatric studies is seen post-2020, peaking in 2024.

As illustrated in Figure 2, the primary evidence base is not only small but also very recent, with all identified studies published from 2016 onward.

### 3.3. tPBM Protocols and Parameters

Table 2 maps the extensive variability in reported PBM parameters and highlights frequent omissions across the included studies and supporting literature, illustrating a profound lack of standardization [26,33,34]. Key parameters such as wavelength, irradiance, fluence, and exposure time varied widely, and reporting was often incomplete. The mapped dimensions of heterogeneity included: wavelength (range: 405–1064 nm), power density (3 mW/cm^2^ to 700 mW/cm^2^), energy density (2 J/cm^2^ to 60 J/cm^2^), exposure time per site (2 s to 40 min), application site (masseter, spine, prefrontal cortex, etc.), and delivery mode (contact vs. non-contact). Frequent non-reporting of parameters such as spot size, duty cycle, and total energy per session was also noted. A critical parameter often under-reported was the light source type (laser vs. LED). Lasers provide coherent, monochromatic light, while LEDs are non-coherent and often have a broader spectral bandwidth. This distinction affects beam characteristics and potential tissue penetration yet was frequently omitted from the reported protocols.

**Mapping of PBM Protocol Parameters and Reporting Heterogeneity**

A critical finding was the frequent presence of internally inconsistent or physically implausible dosimetry reporting, as quantified in the dosimetric analysis presented in Table 3. For example, in Santos et al. (2016) [28], the calculated irradiance (3 W/cm^2^) was 1000× higher than the reported value (3 mW/cm^2^), and the calculated fluence (60 J/cm^2^) was 15× higher than reported (4 J/cm^2^). Such discrepancies compromise replicability and safety assessment.

### 3.4. Spasticity and Functional Outcomes

All five studies reported reductions in spasticity (typically via the Modified Ashworth Scale) and/or improvements in motor function immediately post-intervention. The reported outcome measures included the Modified Ashworth Scale for spasticity and various motor function scales. All studies reported reductions in spasticity scores and/or improvements in motor function post-intervention. The study designs were characterized by an absence of sham controls and blinding (see Section 3.6).

### 3.5. Safety, Tolerability and Neurodevelopmental Considerations

Adverse events were not systematically assessed or reported in any included study. No serious adverse events were reported, but monitoring was insufficient to support safety conclusions. A critical gap identified was the complete absence of pediatric-specific safety or dosimetry protocols. The optical and thermal properties of the developing pediatric skull and brain differ from adults [26], meaning adult-derived dosimetry cannot be assumed safe or effective. None of the studies incorporated pediatric head models or real-time thermal monitoring.

### 3.6. Methodological Gaps and Summary of Key Findings

The methodological mapping revealed the following features across the evidence base: the use of uncontrolled observational or pilot designs (*n* = 5), small sample sizes (total N = 45), absence of sham-controlled or double-blinded trials, and inconsistent reporting of intervention parameters (as mapped in Section 3.3 and Table 3). Adverse event monitoring and pediatric-specific safety protocols were not reported. All studies reported positive directional trends in outcomes.

## 4. Discussion

The mapping conducted in this scoping review reveals a severely limited and fragmented evidence base for tPBM in pediatric CP spasticity. As detailed in the Results and mapped in Table 3, the field is characterized by a profound heterogeneity in reported parameters (e.g., wavelength and irradiance) and frequent internal inconsistencies in dosimetry calculations, which challenge replicability and meaningful synthesis. As detailed in the Results, all included studies reported reductions in spasticity scores post-intervention. Furthermore, outcome measures like the Modified Ashworth Scale (MAS) are unable to distinguish true neural spasticity from other components of muscle overactivity or passive tissue stiffness. Therefore, reported “reductions in spasticity” may reflect a composite effect on neural excitability, muscle visco-elastic properties, or both. Given these profound methodological limitations, alongside the absence of sham controls and blinding, the reported positive outcomes, while biologically plausible given tPBM’s mechanisms [7,8,36], are confounded by non-specific factors and cannot be considered evidence of efficacy. A pivotal insight is that pediatric tPBM protocol development must be fundamentally informed by pediatric-specific dosimetric research [26]. The Castaño-Castaño et al. (2024) study, though not a clinical trial, provides essential empirical data showing that cranial thickness and density, varying with age and condition, are primary determinants of intracranial fluence [26]. This underscores that pediatric tPBM is not a matter of scaling down adult parameters. The profound parameter inconsistencies identified in clinical studies (Table 3) likely represent a fundamental disconnect between applied surface doses and unknown, highly variable intracranial doses.

This review aligns with and extends the findings of the recent systematic review by Jiménez et al. (2024), which also highlighted extreme protocol heterogeneity and high risk of bias [37]. The incremental contribution of this scoping review is threefold: First, it employs a broader mapping methodology to characterize the entire landscape of PBM applications (transcranial and peripheral) for pediatric CP spasticity, not just to assess efficacy. Second, it provides a novel, detailed critical analysis of dosimetric plausibility and internal consistency (Table 3), moving beyond cataloging heterogeneity to questioning the validity of reported doses. Third and most critically, it establishes a dedicated framework for analyzing neurodevelopmental considerations, pediatric-specific safety, and the foundational dosimetric challenges, conceptual domains not deeply explored in prior syntheses. Thus, while confirming the field’s immaturity, this review provides a more detailed map and a distinct set of priorities focused on the pediatric-specific pathway to translation.

### 4.1. Distinguishing Transcranial and Peripheral PBM Mechanisms

This review focused on tPBM, yet most included studies applied PBM peripherally to the muscles or spine. The mechanisms of peripheral PBM (pPBM) are distinct but potentially complementary. pPBM applied to spastic muscles may directly reduce local inflammation, modulate muscle spindle sensitivity, and improve microcirculation, thereby reducing tonic stretch reflex activity. Application to the spine could target the spinal cord circuitry directly, influencing alpha motor neuron excitability and intraspinal processing. While these peripheral effects are plausible for spasticity reduction, they do not address the potential for tPBM to induce supraspinal, cortical reorganization. This mechanistic distinction underscores the heterogeneity in the literature and highlights the need for future studies to clearly define and justify the chosen target (cortical vs. spinal vs. muscular) based on the hypothesized pathophysiology.

### 4.2. Interpretation of Mapped Evidence and Its Implications

The mapping exercise reveals several critical areas requiring attention to advance the field:

The heterogeneity and inconsistencies in parameter reporting (Table 2 and Table 3) highlight a non-negotiable priority: the adoption of a standardized dosimetry reporting checklist in all future publications. Based on this review, non-negotiable research priorities include:

1. **Foundational Pediatric Dosimetry:** A pivot to sham-controlled, dose-finding studies grounded in pediatric-specific computational head models [26,38].
2. **Standardized Reporting Framework:** Mandatory reporting of light source, optical parameters, verified power, beam geometry, delivered dose, application details, and safety protocols.
3. **Pediatric Safety Protocol:** A multi-layered framework including real-time thermal monitoring, ocular protection, structured adverse event diaries, and long-term developmental follow-up overseen by a Data Safety Monitoring Board.
4. **Core Outcome Sets:** Inclusion of functional measures (e.g., Goal Attainment Scaling), patient-reported outcomes (quality of life and pain), and caregiver burden assessments alongside impairment measures like the MAS [39].

**Ethical Framework and Proposed Pivotal Trial Design:** It is ethically reasonable to study tPBM in children because it is non-invasive and has a solid scientific basis, with a generally favorable safety profile in adults. The potential benefits of finding a new treatment that could help reduce their symptoms and improve their lives outweigh the low risks, especially when current standard treatments often come with significant side effects or limitations. The first definitive trial should be a multi-center, triple-blind, sham-controlled, dose-ranging RCT undertaken as follows: Population: 120 children (6–12 years) with spastic CP, GMFCS I-III; Intervention: active tPBM at fluence levels derived from pediatric head modeling, applied to M1; Comparator: a credible, identical-appearing sham device [40]; Protocol: 18 sessions over 6 weeks; Primary Outcome: change in MAS; Key Secondaries: Goal Attainment Scaling, Pediatric Quality of Life Inventory, and safety.

**Clinical Implications:** Given the current pre-preliminary state of evidence, tPBM must be considered an unproven experimental intervention for spasticity in pediatric CP. It should not be implemented in clinical practice outside of rigorously controlled research trials. To move tPBM from “experimental” to “adjunctive clinical option,” the field must produce consistent, replicated evidence from at least two independent, high-quality RCTs demonstrating: (1) a statistically significant and clinically meaningful reduction in spasticity versus sham; (2) a favorable pediatric safety profile; (3) functional translation; and (4) a standardized, reproducible protocol supported by pediatric dosimetry models.

### 4.3. Strengths and Limitations

Strengths include a comprehensive, systematic search across multiple databases, adherence to PRISMA-ScR guidelines, and a novel critical analysis of dosimetric plausibility. Limitations include single-reviewer screening and data extraction (though steps were taken to ensure transparency), and the exclusion of non-English studies and gray literature, which is unlikely to change the fundamental conclusion of a critically underdeveloped evidence base [24].

### 4.4. Neurodevelopmental Considerations: Windows, Plasticity, and Trajectories

A core objective of this review was to explore neurodevelopmental considerations—a dimension notably absent from the empirical literature. The developing brain is not a miniature adult brain; it possesses unique dynamics that must inform tPBM research.

**(1) Enhanced Plasticity and Critical/Sensitive Periods:** The pediatric brain exhibits heightened neuroplasticity, with sensitive periods for motor and sensory system development. tPBM, by potentially boosting BDNF and synaptic efficacy, could theoretically interact with these plastic windows to amplify the effects of concomitant rehabilitation (experience-dependent plasticity).

**(2) Altered Trajectories in CP:** CP involves an early lesion, leading to maladaptive plasticity and altered developmental trajectories (e.g., corticospinal tract reorganization). tPBM’s modulatory effects could aim not merely to suppress spasticity but to guide functional reorganization toward more typical patterns.

**(3) Developmental Dosimetry:** As noted, cranial properties change with age. Neurodevelopmental considerations extend beyond safety to dose–response: the optimal stimulatory dose for a plastic, developing neural network may differ fundamentally from that for a mature, stable one. The complete lack of discussion on these points in the existing studies represents a profound gap. Future protocols must be designed with explicit hypotheses about how tPBM interacts with developmental neurobiology, not just adult pathophysiology.

## 5. Conclusions

This review sought to map the existing evidence on transcranial photobiomodulation (tPBM) for treating spasticity in children with cerebral palsy, particularly concerning neurodevelopmental factors, treatment protocols, functional outcomes, and methodological gaps. The results demonstrate the evidence base remains at a pre-preliminary stage, with few published studies, widespread methodological shortcomings, and no safety protocols designed for children. Although some studies report positive trends, the current literature, marked by inconsistent methods, lack of blinding, and poorly reported dosimetry, cannot yet support efficacy claims or clinical use.

The main value of this review is its clear identification of critical gaps in dosing, safety, and trial design that must be resolved before tPBM can be scientifically validated. Moving forward, research must prioritize well-controlled, sham-blinded trials based on pediatric-specific modeling and standardized safety reporting. Until such rigorous evidence is available, tPBM should remain an experimental intervention confined to research settings.

Future investigations must prioritize sham-controlled, dose-ranging trials with pediatric-specific computational modeling to ensure accurate intracranial dosing. Standardized reporting of photobiomodulation parameters and the development of pediatric safety guidelines are essential to advance the field. Until such robust evidence is generated, tPBM should remain restricted to research settings and not be adopted into routine clinical practice for pediatric cerebral palsy. Clinicians and researchers should collaborate in establishing core outcome sets that integrate both impairment-based and patient-reported measures to better capture the functional and qualitative impacts of intervention.

## Figures and Tables

**Table 1:** Study characteristics: limited and heterogeneous evidence base.

Author (Year)	Country	Study Design	CP Type	Age	Sex Dist.	PBM Target
Dabbous et al. (2022) [30]	Brazil	Longitudinal Observational	30 children with spastic CP • Quadriplegic: 23 (76.6%) • Diplegic: 5 (16.7%) • Hemiplegic: 2 (6.7%)	8–14 years (Mean: 10.1 ± 2.5)	19 Male (63.3%) 11 Female (36.7%)	Peripheral (Masseter & Temporalis Muscles)
Silva et al. (2022) [29]	Brazil	Randomized, Single-Blind Pilot	12 children (CP type not specified)	Not Specified	Not Specified	Peripheral (Spinal Area)
Fernandes et al. (2024) [31]	Brazil	Integrative Literature Review	N/A (Synthesizes primary studies)	N/A	N/A	Peripheral (Masseter Muscle)
Nairuzet al. (2024) [32]	Colombia	Experimental Study	3 participants (Ex vivo/in situ skull analysis, not a clinical CP population)	Not Applicable	Not Applicable	Transcranial (Prefrontal Cortex)
Dompe et al. (2012) [15]	Pakistan	Single, Open, Non-Comparative	10 children (CP type not specified)	Not Specified	Not Specified	Transcranial (“AcuLaser” therapy to scalp)

**Table 2:** Maps the extensive variability in reported PBM parameters and highlights frequent omissions across the included studies and supporting literature, illustrating a profound lack of standardization.

Author	Wavelength (nm)	Power/Avg. Power	Spot Size (cm^2^)	Irradiance (mW/cm^2^)	Energy Density (J/cm^2^)	Exposure Time Per Site	Duty Cycle/Pulse Freq.	Number of Sites	Total Energy Per Session (J)	Application Site	Application Mode
Santos et al. (2016) [28]	808 ± 3 nm	120 mW	4 mm^2^	3 mW/cm^2^	4	20 s	Continuous (CW)	4	2.4 J	Masseter and temporalis muscles on both sides of the face	Contact
Shivappa et al. (2016) [16]	Varying: 665 nm, 730 nm, 810 nm, 980 nm	50 mW	0.78 cm^2^	10 or 20 mW/cm^2^	2	120 s	Continuous (CW)	Not Reported	36 J/cm^2^	Scalp	Contact
Dabbous et al. (2022) [30]	632.8 nm	50 mW	1 cm^2^	63.6 mW/cm^2^	3.0 J	60 s	Continuous (CW)	8	24 J	8 immune acupoints (e.g., ST36, BL18)	Laser acupuncture (direct skin contact)
Silva et al. (2022) [29]	850 nm	500 mW	11.34 cm^2^	176 mW/cm^2^	9 J/cm^2^	50 s	Continuous (CW)	4	100 J	Area without spiny process (4 sequential points)	Contact
Avancini et al. (2024) [35]	635–905 nm (660 nm superficial, 810 nm deep)	200 mW	Not reported	Not reported	3–25 J	30 s–5 min	Continuous (CW)	Not reported	400 J	Varied	Not reported
Nairuzet (2024) [32]	405–1064 nm (e.g., 405 nm GaN, 810 nm GaAs)	27, 100–176, 200 µW (0.027–0.176 W)	~0.708 cm^2^ (9.5 mm aperture)	38.2–… mW/cm^2^	Not reported	Not reported	50%, 100 Hz	25	Not reported	Supraorbital bone (Fp1/Fp2)	Non-contact
Fernandes et al. (2024) [31]	600–1100 nm (810 nm most common)	3.4 W	13.6 or 5 cm^2^	250 mW/cm^2^	60 J/cm^2^ (most common)	20 min (most common)	Continuous or pulsed (10/40 Hz)	97 articles	1632 J (most common)	Prefrontal cortex (most common)	Contact
Fernandess et al. (2024) [31]	808 nm (or 660/808 nm ±10 nm)	120 mW	4 cm^2^	3 W/cm^2^	3 J/cm^2^	20 s	Continuous (CW)	2	2.4 J	Masseter and temporal muscles	Contact
Nairuz et al. (2024) [32]	Multiple: 808, 660, 850, 633, 870, 810, 980, 785, 905, 1064 nm	10 mW/cm^2^–15 W	0.196 cm^2^ (for some)	10–700 mW/cm^2^	1.2–60 J/cm^2^	2 s–40 min	Continuous or pulsed (10/3000 Hz)	Varied	Not reported	Cortex, head, forehead, etc.	Implied external contact
Soe et al. (2025) [22]	630–1064 nm	Not reported	Not reported	Not reported	1–100 J/cm^2^	167 s	50%, 100 Hz	2	5.2–210 J	Various muscle tissues	Not reported
Dompe et al. (2012) [15]	660 nm (body/auricular), 820 nm (scalp)	50 mW (660 nm), 100 mW (820 nm)	~1 cm (body), 1–3 cm (scalp)	Not reported	Not Reported	30 s (body), 45 s (scalp)	Not reported	Not reported	12 J (body), 18 J (scalp)	Body, auricular points, scalp	Contact

**Note**: Light source type is a critical but often under-reported parameter. Based on device descriptions and wavelengths, studies using single, precise wavelengths (e.g., 808 nm and 632.8 nm) typically employed diode or Helium–Neon **laser**. Studies describing broad-spectrum or “light therapy” (e.g., Shivappa et al., 2016 [16]; Avancini et al., 2024 [35]) likely used non-coherent **LED** sources. The review by Fernandes et al. (2024) encompassed both modalities [31].

**Table 3:** Dosimetric analysis and identified inconsistencies in exemplar studies.

Study (Application)	Reported and Calculated Parameters	Inconsistency and Implied Reconciliation
Dabbouss et al. (2022) (Peripheral) [30]	Reported: Power = 120 mW, Spot Size = 4 mm^2^ (0.04 cm^2^), Time = 20 s, Reported Irradiance = 3 mW/cm^2^, Reported Fluence = 4 J/cm^2^. Calculated Irradiance: 120 mW/0.04 cm^2^ = 3000 mW/cm^2^ (3 W/cm^2^). Calculated Fluence (from params): 3000 mW/cm^2^ × 20 s = 60,000 mJ/cm^2^ = 60 J/cm^2^.	FLAG: Critical Mismatch. The calculated irradiance (3 W/cm^2^) is 1000× higher than the reported 3 mW/cm^2^. The calculated fluence (60 J/cm^2^) is 15× higher than the reported 4 J/cm^2^. The reported values would only be plausible if the beam was heavily defocused to a ~40 cm^2^ spot size, which was not stated.
Nairuzet et al. (2024) (Transcranial) [32]	Reported: (810 nm): Avg. Power = 0.176 W, Aperture Area = 0.708 cm^2^, Duty Cycle = 50%, Stated Fluence = 60 J/cm^2^. Calculated Irradiance: 176 mW/0.708 cm^2^ ≈ 249 mW/cm^2^. Time for Stated Fluence: (60,000 mJ/cm^2^)/(249 mW/cm^2^ × 0.5) ≈ 482 s (8 min).	FLAG: Implausible Reconciliation. The stated 60 J/cm^2^ fluence requires an implausibly long ~8 min exposure per site. A typical 30–60 s exposure with these parameters yielded a more credible 3.7–7.5 J/cm^2^, suggesting a significant overstatement of the delivered dose.
Silva et al. (2022) (Peripheral) [29]	Reported: Power = 500 mW, Spot Size = 11.34 cm^2^, Time = 50 s, *Reported Fluence* = 9 J/cm^2^. Calculated Irradiance: 500 mW/11.34 cm^2^ ≈ 44.1 mW/cm^2^. Calculated Fluence: 44.1 mW/cm^2^ × 50 s = 2205 mJ/cm^2^ = 2.2 J/cm^2^	FLAG: Mismatch. The calculated fluence (2.2 J/cm^2^) is 4× lower than the reported 9 J/cm^2^. This discrepancy could only be reconciled with the reported power and time if the actual irradiated area was ~2.5 cm^2^, not 11.34 cm^2^, indicating a potential error in reported spot size or energy density.

## Data Availability

All data generated or analyzed during this study are included in this published article.

## Supplementary Materials

The following supporting information can be downloaded at: https://www.mdpi.com/article/10.3390/brainsci16030272/s1, Table S1: Preferred Reporting Items for Systematic reviews and Meta-Analyses extension for Scoping Reviews (PRISMA-ScR) checklist.
