# Supplementary material for: Transcranial Photobiomodulation for Spasticity in Pediatric Cerebral Palsy: A Scoping Review of Neurodevelopmental Considerations, Treatment Protocols, Functional Outcomes, and Methodological Gaps
Source: Brain Sci. 2026 Feb 28;16(3):272. doi: 10.3390/brainsci16030272 (PMC13024162; doi:10.3390/brainsci16030272)
Supplement: Supplementary file 1 [file brainsci-16-00272-s001.zip › brainsci-4151535-supplementary.pdf]

Preferred Reporting Items for Systematic reviews and Meta-Analyses extension for Scoping Reviews (PRISMA-ScR) Checklist

SECTION	ITEM	PRISMA-ScR CHECKLIST ITEM	REPORTED ON PAGE #
**TITLE**			
Title	1	Identify the report as a scoping review.	**1 (Title):** The subtitle includes "A Scoping Review..."
**ABSTRACT**			
Structured summary	2	Provide a structured summary that includes (as applicable): background, objectives, eligibility criteria, sources of evidence, charting methods, results, and conclusions that relate to the review questions and objectives.	**1 (Abstract):** Contains structured sections: Background, Objective, Methods, Results, Conclusions.
**INTRODUCTION**			
Rationale	3	Describe the rationale for the review in the context of what is already known. Explain why the review questions/objectives lend themselves to a scoping review approach.	**1-2 (Introduction):** Describes the fragmented evidence base for tPBM in pediatric CP and states "A scoping review is the most appropriate methodology..."
Objectives	4	Provide an explicit statement of the questions and objectives being addressed with reference to their key elements (e.g., population or participants, concepts, and context) or other relevant key elements used to conceptualize the review questions and/or objectives.	**2 (Aim and Objectives):** Explicit Aim and four numbered Objectives are provided.
**METHODS**			
Protocol and registration	5	Indicate whether a review protocol exists; state if and where it can be accessed (e.g., a Web address); and if available, provide registration information, including the registration number.	**2 (Methods, first paragraph):** "A pre-defined protocol outlined the objectives, search strategy, and data analysis plan." (No registration info or link provided).
Eligibility criteria	6	Specify characteristics of the sources of evidence used as eligibility criteria (e.g., years considered, language, and publication status), and provide a rationale.	**2-3 (2.1. Eligibility Criteria):** Uses PCC framework (Population, Concept, Context). Specifies age, condition, intervention focus, date range (2000-2025), English language, and rationale for exclusions.
Information sources*	7	Describe all information sources in the search (e.g., databases with dates of coverage and contact with authors to identify additional sources), as well as the date the most recent search was executed.	**3 (2.2. Information Sources...):** Lists eight databases and states search covered studies "from January 1, 2000, to September 30, 2025."
Search	8	Present the full electronic search strategy for at least 1 database, including any limits used, such that it could be repeated.	**3 (2.2. Information Sources...):** Describes search concepts (PBM, CP, spasticity) and keywords/MeSH but does not provide a full strategy for a specific database.

St. Michael's
Inspired Care.
Inspiring Science.

SECTION	ITEM	PRISMA-ScR CHECKLIST ITEM	REPORTED ON PAGE #
Selection of sources of evidence†	9	State the process for selecting sources of evidence (i.e., screening and eligibility) included in the scoping review.	**3 (2.3. Selection of Sources of Evidence):** Describes steps: duplicate removal, title/abstract screening by single reviewer, full-text assessment. Mentions interval between phases and documentation.
Data charting process‡	10	Describe the methods of charting data from the included sources of evidence (e.g., calibrated forms or forms that have been tested by the team before their use, and whether data charting was done independently or in duplicate) and any processes for obtaining and confirming data from investigators.	**3 (2.4. Data Extraction (Charting):** States a standardized form was developed. Mentions key variables extracted. Does not specify independent/duplicate charting. Form is noted as in Supplementary Materials.
Data items	11	List and define all variables for which data were sought and any assumptions and simplifications made.	**3 (2.4. Data Extraction (Charting):** Lists key variables: study/participant characteristics, PBM parameters, outcomes, adverse events.
Critical appraisal of individual sources of evidence§	12	If done, provide a rationale for conducting a critical appraisal of included sources of evidence; describe the methods used and how this information was used in any data synthesis (if appropriate).	**3-4 (2.6. Critical Appraisal...):** States "a formal risk-of-bias assessment was not conducted." Instead, methodological features and reporting quality were mapped.
Synthesis of results	13	Describe the methods of handling and summarizing the data that were charted.	**3 (2.5. Data Analysis and Synthesis):** Describes descriptive numerical summary and narrative synthesis, with data tabulation.
**RESULTS**			
Selection of sources of evidence	14	Give numbers of sources of evidence screened, assessed for eligibility, and included in the review, with reasons for exclusions at each stage, ideally using a flow diagram.	**4 (3.1. Study Selection):** Provides numbers: 345 records, 88 duplicates removed, 257 screened, 228 excluded, 29 full-text assessed, 24 excluded, 5 included. **Figure 1** is the PRISMA flow diagram.
Characteristics of sources of evidence	15	For each source of evidence, present characteristics for which data were charted and provide the citations.	**4-5 (3.2. Characteristics... & Table 1):** Table 1 summarizes key characteristics (author, design, population, PBM target) for the five included studies, with citations.
Critical appraisal within sources of evidence	16	If done, present data on critical appraisal of included sources of evidence (see item 12).	**N/A (Not formally conducted).** Methodological gaps are summarized narratively in 3.6.
Results of individual sources of evidence	17	For each included source of evidence, present the relevant data that were charted that relate to the review questions and objectives.	**5-7 (3.3. tPBM Protocols..., 3.4. Spasticity..., 3.5. Safety...):** Data on protocols, parameters, outcomes, and safety are synthesized across studies in text and Tables 2 & 3. Specific study findings (e.g., Santos et al.) are discussed.
Synthesis of results	18	Summarize and/or present the charting results as they relate to	**7-8 (3.6. Methodological Gaps...):** Provides a narrative

St. Michael's
Inspired Care.
Inspiring Science.

SECTION	ITEM	PRISMA-ScR CHECKLIST ITEM	REPORTED ON PAGE #
		the review questions and objectives.	summary of key findings mapped to the review's objectives (protocol heterogeneity, outcome trends, safety gaps, methodological fragility).
**DISCUSSION**			
Summary of evidence	19	Summarize the main results (including an overview of concepts, themes, and types of evidence available), link to the review questions and objectives, and consider the relevance to key groups.	**8-9 (4. Discussion, first two paragraphs):** Summarizes central finding (severe scarcity/fragmentation), links to objectives (protocols, outcomes, gaps), and discusses relevance (need for pediatric-specific research).
Limitations	20	Discuss the limitations of the scoping review process.	**9 (4.2. Strengths and Limitations):** Lists limitations: single-reviewer screening/extraction, exclusion of non-English studies and grey literature.
Conclusions	21	Provide a general interpretation of the results with respect to the review questions and objectives, as well as potential implications and/or next steps.	**9-10 (5. Conclusions & Implications...):** Interprets the "pre-preliminary stage" of evidence and provides specific implications for future research and clinical practice.
**FUNDING**			
Funding	22	Describe sources of funding for the included sources of evidence, as well as sources of funding for the scoping review. Describe the role of the funders of the scoping review.	**10 (Funding):** States "This research received no external funding." No funding info is provided for the included studies.

JBI = Joanna Briggs Institute; PRISMA-ScR = Preferred Reporting Items for Systematic reviews and Meta-Analyses extension for Scoping Reviews.
* Where *sources of evidence* (see second footnote) are compiled from, such as bibliographic databases, social media platforms, and Web sites.
† A more inclusive/heterogeneous term used to account for the different types of evidence or data sources (e.g., quantitative and/or qualitative research, expert opinion, and policy documents) that may be eligible in a scoping review as opposed to only studies. This is not to be confused with *information sources* (see first footnote).
‡ The frameworks by Arksey and O'Malley (6) and Levac and colleagues (7) and the JBI guidance (4, 5) refer to the process of data extraction in a scoping review as data charting.
§ The process of systematically examining research evidence to assess its validity, results, and relevance before using it to inform a decision. This term is used for items 12 and 19 instead of "risk of bias" (which is more applicable to systematic reviews of interventions) to include and acknowledge the various sources of evidence that may be used in a scoping review (e.g., quantitative and/or qualitative research, expert opinion, and policy document).

From: Tricco AC, Lillie E, Zarin W, O'Brien KK, Colquhoun H, Levac D, et al. PRISMA Extension for Scoping Reviews (PRISMAScR): Checklist and Explanation. Ann Intern Med. 2018;169:467–473. doi: 10.7326/M18-0850.
